# Physical Activity Promotion Tools in the Portuguese Primary Health Care: An Implementation Research

**DOI:** 10.3390/ijerph17030815

**Published:** 2020-01-28

**Authors:** Romeu Mendes, Marlene Nunes Silva, Catarina Santos Silva, Adilson Marques, Cristina Godinho, Rita Tomás, Marcos Agostinho, Sara Madeira, Alexandre Rebelo-Marques, Henrique Martins, Pedro J. Teixeira, Diogo Cruz

**Affiliations:** 1 Programa Nacional para a Promoção da Atividade Física, Direção-Geral da Saúde, 1049-005 Lisboa, Portugal; 2 EPIUnit—Instituto de Saúde Pública, Universidade do Porto, 4050-600 Porto, Portugal; 3 Administração Regional de Saúde do Norte, 4000-477 Porto, Portugal; 4 Centro Interdisciplinar de Estudo da Performance Humana, Faculdade de Motricidade Humana, Universidade de Lisboa, 1495-751 Oeiras, Portugal; 5 Faculdade de Educação Física e Desporto, ULHT, 1749-024 Lisboa, Portugal; 6 Instituto Universitário de Lisboa (ISCTE-IUL), CIS-IUL, 1649-026 Lisboa, Portugal; 7 Portugal Football School, Federação Portuguesa de Futebol, 1495-433 Oeiras, Portugal; 8 Administração Regional de Saúde de Lisboa e Vale do Tejo, 1700-179 Lisboa, Portugal; 9 ISAMB, Faculdade de Medicina, Universidade de Lisboa, 1649-028 Lisboa, Portugal; 10 Faculdade de Medicina, Universidade de Coimbra, 3000-370 Coimbra, Portugal; 11 SPMS—Serviços Partilhados do Ministério da Saúde, 1050-099 Lisboa, Portugal

**Keywords:** physical activity, exercise, primary health care, brief assessment, brief counseling, monitoring

## Abstract

Background: This paper aims to discuss how physical activity (PA) brief assessment, brief counseling, and self-monitoring tools were designed and implemented in the Portuguese National Health Service (NHS), and to report on their current use by health professionals and citizens. Methods: Three digital tools to facilitate PA promotion in primary health care (PHC) were developed: 1) a PA brief assessment tool was incorporated in the electronic health record platform “SClínico Cuidados de Saúde Primários“; 2) a brief counseling tool was developed in the software “PEM—Prescrição Eletrónica Médica” (electronic medical prescription); and 3) a “Physical Activity Card” was incorporated in an official NHS smartphone app called “MySNS Carteira”. Results: From September 2017 to June 2019, 119,386 Portuguese patients had their PA assessed in PHC. Between December 2017 and June 2019, a total of 7957 patients received brief intervention for PA by a medical doctor. Regarding the app “MySNS Carteira”, 93,320 users activated the “Physical Activity Card”, between February 2018 and December 2018. Conclusions: These tools represent key actions to promote PA among Portuguese citizens using PHC as a priority setting. Further initiatives will follow, including proper assessment of their clinical impact and training programs for health care professionals on PA promotion.

## 1. Introduction

According to the new World Health Organization (WHO) Global Action Plan on Physical Activity 2018–2030 [1], effective national action to reverse current trends and reduce disparities in physical activity (PA) requires a “systems-based” approach, grounded on four strategic objectives (create active societies, active environments, active people, and active systems). These may be achievable through several policy actions, including a strategic combination of “upstream” policy actions with “downstream” individually focused approaches (educational and informational). Action 3.2 highlights the need to incorporate PA into health services by implementing systems for patient assessment and counseling aiming to increase patients’ PA and reduce sedentary behavior. It also establishes that assessment and counseling should be made by appropriately trained healthcare providers in every single opportunity, in primary and secondary health care, as part of universal health care, ensuring community and patient involvement. In this regard, and as proposed actions for member states, there is a call for the development and implementation of national standardized protocols on patient assessment and brief counseling, adjusted to the local context and culture, as well as resource availability. The need for these actions was already highlighted on the Physical Activity Strategy for the WHO European Region 2016–2025 [2].

Health care systems, namely primary health care (PHC) systems and staff, have a crucial role in PA promotion for several reasons. Firstly, there is a well-established benefit of regular PA in the prevention and treatment of many clinical conditions (especially noncommunicable diseases) and also in promoting physical and mental wellbeing [3,4]. Secondly, health care professionals are respected experts and authorities in matters related to health and are considered reliable sources of information and advice (e.g., advice on PA from medical doctors can be a strong cue to becoming more active) [5]. Finally, and most importantly, PHC staff have frequent contacts with large segments of the population, especially those with poorer health, lower socio-economic status, and/or who are older [6]. This holds a large potential for health care professionals to decisively influence public health outcomes. 

Assessing a patient’s PA level and providing information and brief counseling through strategies that will assist patients in behavior change is crucial [7]. Therefore, there has been a call for PA assessment to be considered a “vital sign” for health [8]. In the traditional sense, vital signs are measurements that reflect basic physiological functions and health status and are used to monitor or screen for health problems. This concept recently evolved to accommodate other types of vital signs equally informative and with important effects on health outcomes (e.g., body mass index, smoking status, alcohol intake, pain level, etc.). PA assessment can provide a valuable insight into patients’ health status and lead to important intervention opportunities [5]. Despite all the evidence and calls to include PA assessment and promotion in the standard of care, challenges remain on how to accomplish this in a way that engages both clinicians and patients [5]. For example, in a recent study, only 36% of American adults that had received health care in the past 12 months were told by a health care professional to increase their PA levels. However, 87% of medical doctors reported that they frequently advised patients to be physically active [9]. In Portugal, in a representative sample of the adult population, 62% of those who had a medical appointment in the last two years reported to have been asked about PA, and 41% had been given advice to increase PA practice by their medical doctor [10]. For 76%, PA was an important health behavior, but only 2% knew the WHO PA recommendations [11], and only 43% recognized daily activities as PA [10].

In order to increase and generalize PA counseling and improve its quality, we need to build upon the science of behavior change. For example, interventions should be theory and evidence-based, and supported by advanced tools for measuring and influencing behavior, with a potential for broad reach dissemination [12]. Systematic reviews and meta-analyses [13] have highlighted several constructs for which there is good and consistent evidence of positive adherence effects, including intrinsic motivation and enjoyment, self-efficacy, and expected outcomes. Behavior change techniques that effectively promote PA have been identified, and evidence supports the importance of using goal setting and self-monitoring of behavior in counseling [14]. The importance of a person-centered and autonomy-supportive counseling setting has also been stressed as an important aspect in order to maintain behavior over time [14].

The evidence and guidelines for PA behavioral change also point to the potential effectiveness of brief interventions in PHC [15,16]. Evidence suggests that brief advice by clinicians may improve short and long-term engagement with active lifestyles [17,18]. Specifically, clinician counseling as brief as one to five minutes may result in a significant increase in patients’ PA levels. However, although PHC represents an important avenue for PA promotion, effective PA counseling is still hampered by limited time constraints and concurrent tasks [19]. 

In this regard, recent systematic reviews have summarized enabling and reinforcing factors to optimize the effectiveness of PHC PA brief counseling [15,20]. Although brief interventions in PHC showed a small to moderate positive effect on increasing PA levels, better results were obtained by interventions that included i) the use of valid (and multiple) behavior change methods, namely behavioral, cognitive, and motivational approaches such as setting clear, simple, realistic and proximal goals instead of distal ones; emphasizing internal instead of external motivating factors; improving self-efficacy; and use planning and self-monitoring, social support, and follow-up prompts; ii) individual assessment of the needs, motivation, current habits, preferences, and barriers of the patient and establishment of action plans according to this information; iii) use of patient-centered and motivational interviewing principles to anticipate barriers and discuss solutions; and iv) existence of training and resources to carry out such counseling [21].

The United Kingdom National Institute for Health and Care Excellence (NICE) guidelines also emphasize that, in order to improve clinicians’ counseling effectiveness, it is necessary to improve their motivation to increase the quantity and the quality of service provided [22]. One way to achieve this is to decrease the time and increase the resources available to deliver this counseling and include evidence-based behavior change methods as highlighted before. One contemporary framework for promoting internal motivation for changing health behavior is motivational interviewing [23]. It reflects a collaborative, person-centered, and goal-oriented method of communication with particular focus on the “language of change”. It is designed to strengthen an individual’s motivation for a specific goal by eliciting and exploring the person’s own arguments for change. It is theory-consistent [24], considered an evidence-based method [25], and there are currently numerous training opportunities as well as resources (e.g., online networks and manuals) available for interested health professionals. 

A second strategy that could improve counseling effectiveness is to create digital tools, which can be valuable resources to healthcare professionals, minimizing time and energy necessary to give advice and follow-up. Action 4.3 from WHO’s Global Action Plan on Physical Activity 2018–2030 [1], reflecting on the need to create active systems, calls for national and institutional research to stimulate the use of digital technologies and innovation to accelerate the development and implementation of effective policy solutions aimed to increase PA and reduce sedentary behavior.

Regarding technology, exploring the features of smartphones and other mobile devices seems one of the most promising strategies to promote PA either through the monitoring of daily levels and tasks, or by engaging the user in PA challenges and exergames [26,27,28].

This paper aims to discuss how PA brief assessment, brief counseling, and monitoring tools were designed and implemented in the Portuguese National Health Service (NHS), and also reports on its current use by health professionals and citizens.

## 2. Materials and Methods

Within the scope of the Portuguese National Physical Activity Promotion Program, the Directorate-General of Health, in collaboration with the Shared Services of the Ministry of Health, developed three digital tools for the promotion of PA, specifically designed for the PHC of the NHS.

### 2.1. Physical Activity Brief Assessment Tool

The routine assessment of PA (and sedentary behavior) in the health system is the basis for the surveillance of physical inactivity as a risk factor and should be an important starting point for PA brief counseling [29].

**Figure 1:**
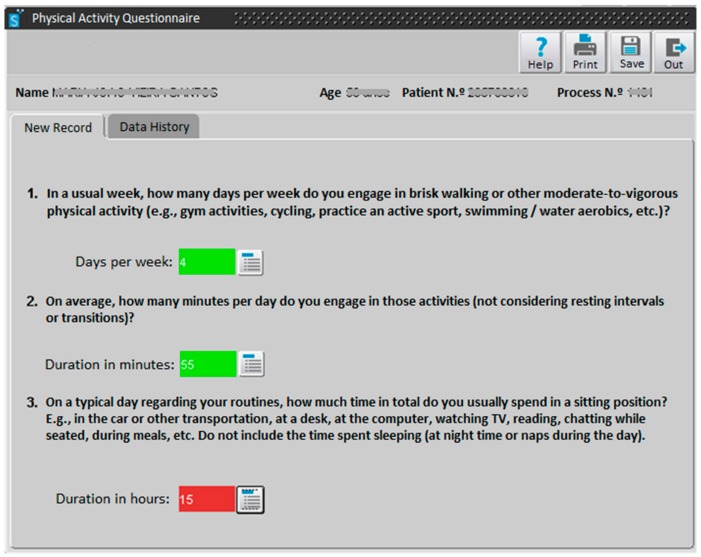
Physical activity brief assessment tool in the database/software “SClínico Cuidados de Saúde Primários”, with three questions.

A tool allowing the assessment of weekly moderate-to-vigorous PA (min/w) and daily sitting-time (h/d) was implemented in the database/software “SClínico Cuidados de Saúde Primários“ (Figure 1) in September 2017. This platform is used as the electronic medical record in the PHC setting to track vital signs, code health problems, and support/record consultations with different health professionals. It is currently used in 300 PHC institutions by 13,000 health professionals.

In order to assess weekly PA level of adults and older adults, two questions are available:

1. In a usual week, how many days per week do you engage in brisk walking or other moderate-to-vigorous physical activity (e.g., gym activities, cycling, practice an active sport, swimming / water aerobics, etc.)?
2. On average, how many minutes per day do you engage in those activities (not considering resting intervals or transitions)?

A “traffic light” feedback system was included to support health professionals and facilitate the interpretation of the results based on the WHO PA recommendations for adults [11]: when the product of both answers is zero, the answer box turns red; when the product is between 1–149 min/w, the box turns yellow; and when the product is equal to or higher than 150 min/w, the box turns green.

In order to assess daily sitting time, a third question is available:

3. On a typical day regarding your routines, how much time in total do you usually spend in a sitting position (e.g., in the car or other transportation, at a desk, at the computer, watching TV, reading, chatting while seated, during meals, etc.)? Do not include the time spent sleeping (at night time or naps during the day).

Again, a “traffic light” feedback system was also included, with the box turning red if the answer is 7 or more h/d, yellow if it is 4-6 h/d, and green if it is 0–3 h/d [30].

The questions are similar to the ones included in the WHO’s Global Physical Activity Questionnaire [31] and are used in several PA questionnaires and surveillance systems. Given that the tool has a “Data History” system (see Figure 1), health professionals can track changes in PA levels. Thus, more than the record of PA levels per se, this tool seeks to assist clinicians and patients to be more aware of PA patterns over time—addressing the possibility of change (i.e., becoming (or not) more active and less sedentary). This assessment is indeed the cornerstone of the brief counseling process. At every encounter/opportunity, even when time is very limited, it is mandatory to assess PA. It is not just for the purpose of monitoring or assessing progress; it has value in its own right. Asking the question(s) “opens the door” for fostering the patient’s reflection and self-awareness. Subsequent intervention can vary, depending on the available time and resources.

Currently, only three types of health professionals can use this PA assessment tool: medical doctors, nurses, and registered dietitians and nutritionists.

### 2.2. Physical Activity Brief Counseling Tool

**Figure 2:**
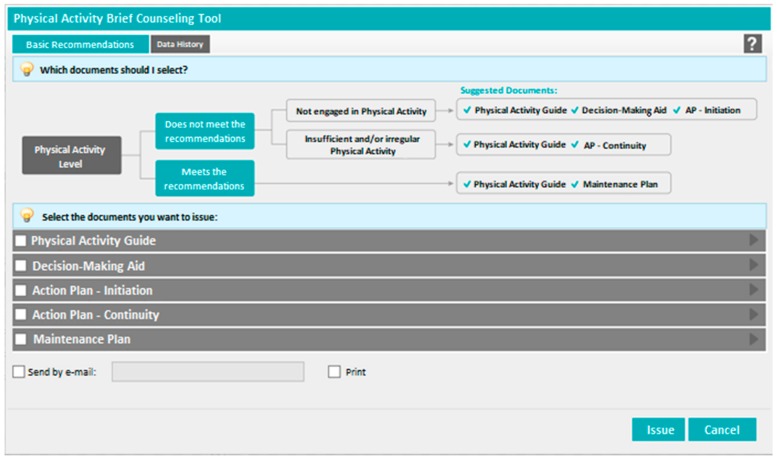
Physical activity brief counseling tool in the software platform “PEM—Prescrição Eletrónica Médica”.

In order to support the establishment of a brief counseling system for PA promotion, a digital tool was developed in the “PEM—Prescrição Eletrónica Médica” (Electronic Medical Prescription; Figure 2) software platform and made available in December 2017. This software is accessible to all medical doctors in Portugal for the prescription of pharmacological treatments, home respiratory care, and other medical devices. In December 2017, from a total of 57,922 medical doctors registered in Portugal, 29,954 were using this software, mainly in the NHS.

The new digital tool consists of five inter-related documents, “guides”, that can be delivered to patients (printed or by email; each guide has one page), according to their current motivation and PA levels. It was developed on the basis of solid scientific evidence, namely on principles of motivational theory [24] and validated behavior change techniques such as goal setting, action planning, coping planning, and self-monitoring [32]. They were also designed to be self-explanatory so that they can be used and explored autonomously without consuming much consultation time. These documents aim to improve the quality of brief counseling intervention and save the clinician’s time and effort.

The five guides, adapted to the level of readiness and PA of the patients, have been developed to support the motivational and self-regulatory processes involved in the adoption of more active lifestyles. One of the guides, the “Physical Activity Guide”, was developed to be a key resource, including important information regarding the PA recommendations for adults and easy-to-implement strategies to reduce sedentary behavior and become more active in everyday life. Moreover, it challenges the patient to identify PA benefits he/she considers to be self-relevant. The information contained in this guide is deemed important for everyone, irrespectively of their current PA levels. PA literacy is still scarce, as previously mentioned, as only 2% of the Portuguese population know the recommendations for PA, and only 43% of the population recognize daily activities as PA [10].

Although knowledge is unquestionably a first step towards behavior change, it is known to be insufficient for sustained behavioral changes [33,34]. Therefore, this guide was designed to be used along with other guides.

For inactive patients who do not yet consider becoming physically active, two other guides have been developed: “Decision-Making Aid” and “Action Plan—Initiation”. The first guide aims to help the patient identify relevant reasons for becoming more physically active and think about barriers and facilitators to engage PA in their daily routine. The second guide helps set goals and define an implementation strategy (i.e., defining the type of activity, where, at what time, with whom, etc.), and it was designed to be used when the individual is already inclined to increase their PA levels.

In patients where PA is not yet a habit but they have already some activity (irregular or insufficient), a guide was developed to support the development of a PA routine and maintained engagement: “Action Plan—Continuity”. The aim of this guide is to foster more effective planning, including the anticipation of potential barriers and establish strategies to better cope with them.

Finally, for patients who already meet the current PA recommendations, a set of suggestions are made in order to help keep up the interest in being active, such as the use of upgraded self-monitoring strategies (i.e., including other types of indicators besides behavior such as emotions during/after practice), challenging oneself to try new activities, change practice scenarios, and becoming a PA “leader”: “Maintenance Plan”.

In order to help select the most appropriate guide(s), a decision algorithm, following the explanation given above, was developed (Figure 2)—indicating the guides to be given to the patient according to the current level of PA and readiness for change—and has been included in the digital tool menu. If the patients does not meet PA recommendations, and they are ambivalent or not interested in starting, the “Decision-Making Aid” (plus the “Physical Activity Guide”) is suggested, followed by the “Action plan—Initiation”. For patients that, despite being motivated and willing to be physically active, do not entirely meet the WHO criteria because of irregular and/or insufficient levels of PA, the “Action Plan—Continuity” (plus the “Physical Activity Guide”) is suggested. Last, and as already mentioned, the “Maintenance Plan” (plus the “Physical Activity Guide”) it is targeted to patients that already meet the current PA recommendations. To facilitate implementation, an additional resource was developed and is available in the Directorate-General of Health’s PA website. This manual contains evidence-based suggestions on how to facilitate the use of brief counseling guides during the medical consultation, depending on the patient’s PA level and time available. This is important because, although the guides can be used autonomously, their effectiveness can be increased when they are facilitated by the healthcare professional in a motivational environment that is compatible with the development of autonomous motivation [20,35].

### 2.3. Physical Activity Self-Monitoring Tool

The smartphone application “MySNS Carteira” was launched in January 2017 in order to include the electronic version of several identification documents/cards used in the health sector such as the NHS card, the immunization card, the allergies card, the living will document, and pharmacological treatments and prescriptions. This app is available as a free download in the *App Store* and in the *Google Play*.

**Figure 3:**
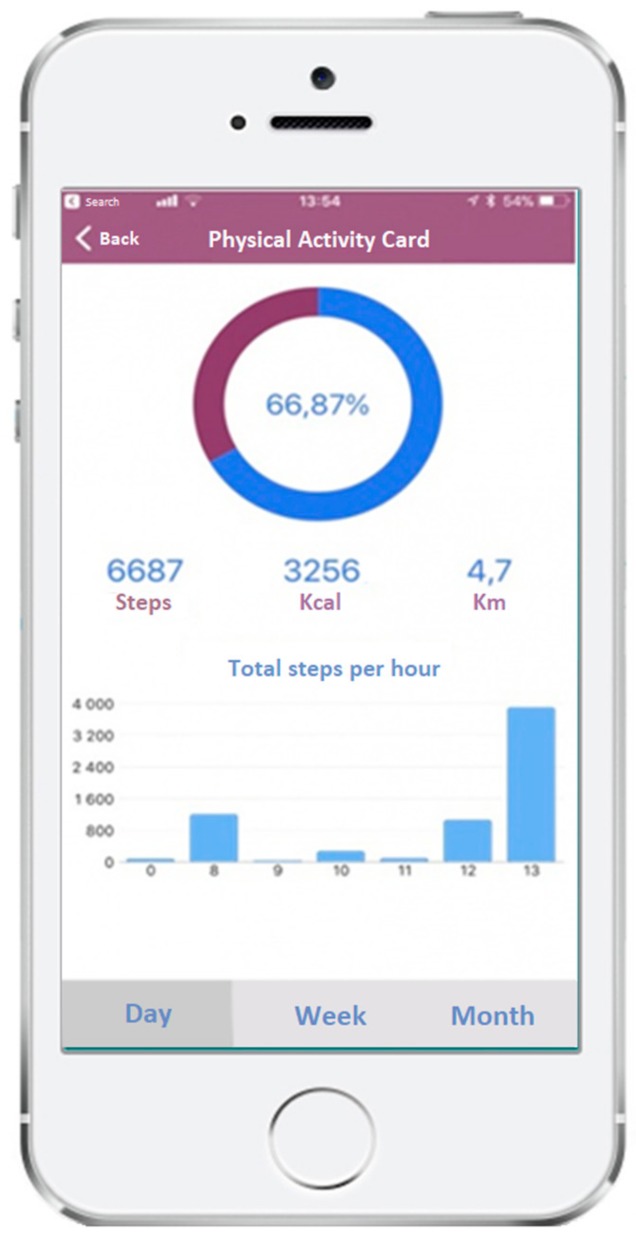
“MySNS Carteira” app—“Physical Activity Card”.

A new feature, the “Physical Activity Card”, became available in February 2018 allowing users to monitor their own PA level. This tool reads the smartphone’s accelerometer information and provides reports (daily, weekly, and monthly) based on the number of steps, distance (km), and energy expenditure (kcal), giving feedback to the user according to international standards (Figure 3).

It is important to note that tools depicted in Figure 1, Figure 2 and Figure 3 were developed to be used in combination. In the scope of PHC consultations, health professionals should routinely conduct a brief assessment of PA and sedentary behavior (tool 1) and, following this assessment, discuss the pattern presented (i.e., increase vs. decrease of PA and sedentary behavior) using also the brief counseling resources (inclosed in tool 2). These resources recommend the use of self-monitoring devices (in order to track the goals and action plans set), and thus, the self-monitoring tool (tool 3) can be prompted at every consultation. Health professionals can ask patients if they are already using the “Physical Activity Card” to monitor their levels of PA, and can also analyze and discuss with patients the data presented by the smartphone application, such as the recommended daily cut-off for the number of steps, or how to interpret the distance (km) and energy expenditure (kcal). This interaction can be crucial for tackling lack of adherence in the follow-up of PA levels.

### 2.4. Ethics Procedures

This was work approved by the Ethics Committee of the Faculdade de Motricidade Humana, Universidade de Lisboa, with the reference CEFMH 03/2019.

## 3. Results

From September 2017 to June 2019, 119,386 patients from the Portuguese PHC system had their PA assessed through the “SClínico Cuidados de Saúde Primários” platform. This number represents a proportion of 1736 per 100,000 users of the NHS (6,876,364 Portuguese citizens). These records were completed by 6183 PHC professionals: 3715 medical doctors (60%), 2382 nurses (39%), and 86 registered dietitians and nutritionists (1%).

Between December 2017 and June 2019, a total of 7957 patients received PA brief counseling using the “PEM—Prescrição Eletrónica Médica” PA guides, meaning that 94 per 100,000 residents in Portugal 15 years or older were reached. A total of 20,494 guides were delivered, the majority in paper format (less than 5% were sent by email). 

Regarding the app “MySNS Carteira”, 93,320 users activated the “Physical Activity Card” between February 2018 and December 2018.

## 4. Discussion

The assessment of PA levels and the provision of brief counseling by health professionals are key actions recommended by both the WHO and the European Commission to promote health-enhancing PA at the country level [2,36,37].

The digital tool that has been developed for assessing PA in every encounter/opportunity is already available in the large majority of PHC units in the country (i.e., the ones using “SClínico Cuidados de Saúde Primários” platform). A full national coverage of this software is expected by the end of 2020. This will enable the use of this tool by all health professionals working in PHC. Furthermore, other healthcare professionals besides medical doctors, nurses, and registered dietitians and nutritionists (e.g., psychologists and physiotherapists) are expected to have access to the system in the near future. PA assessment for children and adolescents with new PA questions and appropriate cut-offs (i.e., 60 min of moderate-to-vigorous PA per day) with immediate feedback is already being prepared, and the expansion of this tool to the Portuguese NHS hospital network within the platform “SClínico Hospitalar” is also under development.

The digital guides that facilitate the delivery of brief counseling are already implemented at full scale, across the country, through the “PEM—Prescrição Eletrónica Médica” system and are available for all medical doctors registered in Portugal. These guides were also made available to other healthcare professionals through Directorate-General of Health’s website, allowing a more “open” access (not restricted to only users that have access to “PEM—Prescrição Eletrónica Médica”), facilitating the scaling up of PA promotion and brief intervention to other health care settings (e.g., occupational medicine).

Because of the aging index of the Portuguese population, another challenge is to increase the percentage of guides sent by e-mail, saving resources and consultation time.

Future developments of the “Physical Activity Card” of the “MySNS Carteira” will include linking its data to the “SClínico Cuidados de Saúde Primários” platform (after patients’ consent), allowing healthcare professionals to monitor their patients regarding their daily PA. Technology literacy is being approached at a national level in order to increase the use of these tools.

Another crucial step concerns training health care professionals. Specific training will be needed to help them acknowledge the role of PA in the prevention, treatment, and management of a vast array of diseases and also understand the importance of assessing current PA levels and motivating their patients to engage in a more active lifestyle. Another relevant area of training is facilitating the use of brief counseling tools in order to maximize their impact and support in sustained behavior change, including training in person-centered motivational strategies and skills (e.g., motivational interviewing). This will be accomplished by integration in undergraduate medical curricula, as well as providing specific postgraduate training on PA promotion and continuing education (including peer-to-peer education facilitated by a network of trained and skilled specialists) for different health care professionals regarding the assessment and brief counseling on PA promotion.

Finally, in order to identify factors that can facilitate and improve the implementation and use of the developed tools, as well as assessing their effectiveness and cost-effectiveness, pilot projects will be conducted in 13 PHC units across different regions of the country. These pilot projects will compare patients receiving brief counseling for PA (including the assessment of PA) with those who do not receive it in relation to various health outcomes. Outcome measures will be assessed both at baseline and follow-up and include the level of PA and sedentary behavior (through self-report and use of objective measures), blood pressure, body mass index, health-related quality of life, medication use, and number of acute medical appointments. In addition to these indicators, qualitative methods—focus groups with patients and interviews with healthcare professionals—will also be used to assess the acceptability of the tools from both perspectives as well as implementation fidelity.

## 5. Conclusions

Portugal has taken a decisive action to promote PA in its citizens using PHC as a priority setting. These initial steps are currently being evaluated and should be followed up by further initiatives, including the provision of solutions for patients in need of supervised exercise programs. Together, these actions will certainly contribute to improve the overall health and quality of life of the population and the sustainability of the health system.
